# Study the forest, not only the trees: Environmental exposures, not genomes, generate most health disparities

**DOI:** 10.3389/fgene.2022.817899

**Published:** 2022-08-19

**Authors:** Taylor V. Thompson, Katherine C. Crocker

**Affiliations:** ^1^ Genetics Department, Albert Einstein College of Medicine, New York, NY, United States; ^2^ Biology Department, University of Puget Sound, Tacoma, WA, United States

**Keywords:** genomics, environmental exposure, health disparities, environmental racism, health equity

## Abstract

As sequencing and analysis techniques provide increasingly detailed data at a plummeting cost, it is increasingly popular to seek the answers to medical and public health challenges in the DNA sequences of affected populations. This is methodologically attractive in its simplicity, but a genomics-only approach ignores environmentally mediated health disparities, which are well-documented at multiple national and global scales. While genetic differences exist among populations, it is unlikely that these differences overcome social and environmental factors in driving the gap in health outcomes between privileged and oppressed communities. We advocate for following the lead of communities in addressing their self-identified interests, rather than treating widespread suffering as a convenient natural experiment.

## Introduction

Genomics is a powerful tool, and as sequencing costs plummet, increasingly popular across research and applied fields. Such popularity does not guarantee equitability in accessibility or application. Many genetic studies have fallen into the same trap as much of western medicine, focusing on the DNA of northwestern Europeans, although little biological reason for this focus has been presented. For example, between 2005 and 2020, 88% of GWAS studies presented findings using sequences from individuals of European ancestry, and 72% reported discoveries using sequences from only the United Kingdom, US, or Iceland (Mills and Rahal, 2019). Such an asymmetry in focus has resulted in calls for more equitable focus in genetic research to reduce the perception of the white European genome as the normal, healthy state. Our colleague Dr. John M. Greally has described the current status of the field of human medical genetics as facing “a balance between ignoring whole sections of the globe, and exploiting them,” noting that “currently, we fall far to the side of ignoring” (JM Greally, personal communication). This disparity in degree of medical research effort between marginalized racialized people and white racialized Europeans and those descended from them mirrors national and global health disparities. However, genomics as a mechanism for addressing population-level health disparities (that is, differences in the burden of health insults) has serious limitations.

## Environment and genetics interact to produce health

Population-level health disparities are affected by both genetics and environmental exposures. A much-discussed 1975 study concerning deleterious multi-generational effects of environmental exposures focused on the descendants of individuals who survived the Dutch Hunger Winter (Stein and Susser, 1975). While the methodology and scientific conclusions of studies on this phenomenon are debated (Paneth and Susser, 1995; Schulz, 2010), the analytical approach of considering environmental determinants of health is sound. It is generally agreed that famine and other adverse environmental factors were drivers of long-term health challenges, not any genetic predisposition of Dutch individuals. Subsequent work has generally demonstrated that environment has profound effects on health (Emeny et al., 2021), yet disparities between Black and Indigenous communities and white communities are frequently assumed to be genetic in origin (effectively blaming the DNA of Black and Indigenous communities), rather than a consequence of environmental racism (Yudell et al., 2016; Borrell et al., 2021).

Global-scale evidence indicates that environmental, not genetic, explanations are strongly implicated in population-level health disparities. For example, life expectancy is a common public health measure (Roubal et al., 2021), and despite a fairly constant genetic background, it shifts dramatically based on environment (The World Bank, 2019a; The World Bank, 2019b). For example, immigrants to the United States from nations with lower life expectancies than the United States experience increased life expectancy beyond the average US-born individual, though their US-born descendants do not (Argeseanu Cunningham et al., 2008; Mehta et al., 2016; Bastian et al., 2020). This pattern is incompatible with a genetic explanation, especially in the presence of the dramatic environmental shifts that accompany immigration.

In the lands lately known as the United States, racialized minoritized people experience disproportionately high levels of direct and indirect environmental exposures (Mikati et al., 2018; Rubio et al., 2020). Acknowledging this reality can only strengthen genomic studies designed to improve health in our communities. For example, a study by Rastogi et al., in 2013 used a study design which matched cases and controls not merely concerning age, sex, and ethnicity, but also drawing from the same geographic area and hospital systems (Rastogi et al., 2013). Such a detailed study design can account for environmental exposures associated with geographic location (e.g., vehicle emissions, heavy metal exposure, water quality) and personal experiences of medical racism in a particular hospital system: such awareness is powerful for building health disparity-related genomics studies. Rigor is gained in the analysis of data beyond sequences, especially those data which are associated with environmental exposures relevant to the condition studied.

Environmental exposures are particularly relevant to health disparities because the prevalence of environmental exposures in a particular community may, if unexamined, result in the mistaken assumption that genetic predisposition is the only, or even primary, cause of observed disease. For example, the impacts of chronic community exposure to lead in drinking water may be misattributed to genetic factors if it generates similar symptoms (e.g., cardiovascular disease and reduced attention span) in multiple generations (see: Levallois et al., 2018). In fact, inaccessible resources, as well as exposures to environmental pollution and the psychological trauma associated with racism and targeted violence have detrimental short- and long-term effects on health of communities on the losing end of health disparities (Richardson and Norris, 2010; Ray, 2021). Interpersonal exposure-mediated health challenges are particularly key to address because they are “sticky” (that is, not confined to a single environment): visibly and hypervisibly racialized people experience racism in effectively all social and geographic locations (Negrete Alfaro, 2011; Linley, 2018; Niles et al., 2020).

## Discussion

Environmental exposures are the results of government policies and social practices, not endogenous biological processes (Shavers and Shavers, 2006). While the function of a DNA sequence cannot be divorced from its environment, neither can the sequence itself be used as a proxy for its environment. In other words, it is impossible for any genome to produce perfect health when immersed in an acutely unhealthy environment. With the increase in computational technology and infrastructure, it is now feasible for analyses to consider not just a DNA sequence, but myriad related variables. Indeed, in failing to do so, we are systematically excising a large proportion of relevant, high-dimensional data and thereby reducing the rigor of our work (Yearby, 2020; Harrison, 2021).

Determining which environmental variables are relevant requires a degree of expertise in community experience that is frequently absent in researchers, who are frequently outsiders. However ignorant we researchers may be, community members have detailed and long-lived expertise concerning their environments and their individual and collective health: recognizing this expertise is key to addressing health disparities, especially those of racialized and marginalized communities. Unfortunately, many of these communities have suffered extensive harm at the hands of researchers working in the name of improving health, access to resources, and human knowledge. To achieve collaboration with these communities, extra measures are necessary to safeguard community autonomy and bolster the right to withdraw consent (Zahara, 2016). Further, community-identified priorities must be recognized as the only legitimate guiding principles for research initiatives in that community. For example, the Strong Heart Study (strongheartstudy.org), an epidemiologic study of cardiovascular disease in American Indians, uses a multi-stage community consent process via which researchers apply to use tribal data for a specifically parameterized study. If consent is granted, at the conclusion of their study, the research team communicates their findings to the communities as part of their application for the communities’ consent to publish that work.

In planning studies concerning the genetics of racialized and marginalized communities, researchers must exercise trust of community members, and just as importantly, skepticism of our own processes and assumptions. In other words, communities are not study systems, to have careers built on their ongoing suffering. It is very probable that our investigations will not find that more molecular research is needed. For example, if an environmental exposure is skewed towards people with presumed (see: Yudell et al., 2016) genetic backgrounds that deviate from the European “standard”, exogenous (e.g., social, governmental) causes are implicated: in this case the path towards health equity lies in social and governmental change, not further genomic research. Only in the case of an evenly distributed exposure which disproportionately harms members of distinct communities, are molecular and genetics studies implicated as a potential source of solutions. And even in this case, it should be considered whether chronic environmental exposures may be more likely drivers than the genetic (Levallois et al., 2018). Genomic studies cannot overcome persistent engineered environmental effects.

In conclusion, we emphasize that mobilizing genetics research to serve oppressed and marginalized communities is not incompatible with ameliorating already-identified sources of harm. However, using genetics as a tool in moving towards a more equitable future requires substantial front-end labor to build structures which protect community autonomy and robust consent (Zahara, 2016). Many necessary changes are disincentivized in current academic and applied research contexts because they would increase the cost of research in terms of time, personnel, or resources. Nevertheless, we call for structural, contextual, and methodological changes in the way that health disparities and genomic research equity are approached. We advocate for following the lead of communities in addressing their self-identified interests, rather than treating widespread suffering as a convenient natural experiment. Until researchers recognize that health disparities are problems to be urgently addressed (often through non-research means), rather than opportunities for career advancement, health equity will remain merely aspirational.

## Data Availability

The original contributions presented in the study are included in the article/supplementary material, further inquiries can be directed to the corresponding author.
